# Social Isolation Causes Behavioural Abnormalities and Reduces Synaptic Proteins in the Brains of *Drosophila*

**DOI:** 10.3390/biology15191721

**Published:** 2026-09-30

**Authors:** Gurlaz Kaur, Heba Mahmood, James Thompson-Dick, Anna Phan

**Affiliations:** 1 Department of Biological Sciences, University of Alberta, Edmonton, AB T6G 2R3, Canada; 2 Neuroscience and Mental Health Institute, University of Alberta, Edmonton, AB T6G 2R3, Canada; 3 Women and Children’s Health Research Institute, University of Alberta, Edmonton, AB T6G 2R3, Canada

**Keywords:** loneliness, mental health, dopamine, synapses, social proximity, clustering, physical activity

## Abstract

Social isolation is known to cause a variety of behavioural abnormalities in many different species. Here we perform a detailed behavioural analysis of how chronic social deprivation alters behaviours in the fruit fly, *Drosophila melanogaster*, within an arena. We identify specific, quantifiable measures to capture behaviours of interest using the previously established cTrax and MATLAB BehavioralMicroarray toolkits. Socially isolated *Drosophila* exhibited behavioural changes in almost all measures assessed, identifying a surprisingly wide range of behavioural deficits. We observe that both male and female isolated flies demonstrate reduced locomotion (walk distance and speed), are more avoidant of other same-sex individuals (increased social distancing), and show reduced clustering into same-sex groups. Because these measures of sociality are assessed in same-sex groups, they are independent of sexual motivations. For the majority of measures taken, we observe the same behavioural changes in isolated male and female flies. In the brain, we observe that social isolation leads to a reduction in synaptic proteins specifically within dopamine neurons, as well as across the whole brain in both sexes, consistent with the varied behavioural abnormalities observed upon social isolation.

## Simple Summary

People who are lonely or socially isolated experience higher rates of anxiety, depression, and dementia. Although profound behavioural changes have been associated with social deprivation in both people and in animal studies for decades, how isolation changes the neurons in the brain to alter behaviour remains a mystery. In this study, we identify several social and non-social behaviours that are altered upon chronic social isolation in *Drosophila*, which can be used to examine how social isolation affects the brain. Moreover, we also discover that a lack of social interaction reduces the amount of synaptic proteins in dopamine neurons and across the entirety of the *Drosophila* brain. This suggests that a lack of social experience leads to the reduced ability of neurons to communicate in a wide range of neural circuits in the brain, possibly leading to the behavioural changes observed upon social isolation.

## 1. Introduction

People who self-report high rates of loneliness or social isolation experience significantly higher rates of depression, dementia, and anxiety [1,2,3,4,5,6,7]. Loneliness is the subjective feeling of isolation, while social isolation is the objective lack of social interactions [8]. While these two measures are not always congruent for an individual, both are known to significantly negatively impact both psychological and physical health. Although it has been known for quite some time that loneliness and social isolation lead to negative health outcomes, the mechanisms by which this occurs remain a mystery [9,10].

Social isolation, in humans and in other animals, is known to cause a wide range of behavioural abnormalities. Several of these behavioural changes appear to be consistent across different species. For example, isolation in humans, rodents, and *Drosophila melanogaster* all decrease sleep [11,12,13,14,15,16] and result in abnormal social behaviours [17,18,19], including increased aggression [20,21,22,23,24,25,26,27,28]. Interestingly, isolated flies also exhibit a reduced lifespan [29], an observation consistent with the higher mortality rates reported in isolated and/or lonely people [9,10].

An increasing number of studies have started to examine the neurobiology of social isolation using the genetic model organism *Drosophila melanogaster* [19,30]. *Drosophila* are sociable; they prefer social to non-social stimuli, spontaneously cluster into social groups, demonstrate collective and cooperative behaviours related to foraging, feeding, and circadian rhythm entrainment, and demonstrate social learning (mate copying and egg-laying behaviour) [31,32,33,34,35,36,37,38,39,40]. Thus, in combination with the advancements in mapping the neural connectome and genetic tools available for this organism [41,42,43], *Drosophila* can be used to make significant advancements towards understanding the mechanisms encoding the experience of social isolation. For example, previous RNA-sequencing on fly brains revealed chronic social-isolation-induced sleep disturbances appear to be related to changes in expression levels of starvation-related genes, which also accounts for increased feeding behaviour in the isolated flies [12].

Several social behaviours are reported to be altered in isolated flies. These include increased aggression that may result from alterations in neuropeptide expression [28], decreased social approach or proximity with other same-sex flies [18,40,44,45,46], and reduced courtship success [47]. Some evidence also suggests that the dopaminergic system might be dysregulated in isolated flies, since isolation reduces dopamine concentrations in the brain [16,40] and social proximity also appears to be dependent on the proper functioning of the dopamine system [46,48].

In this study, we perform a detailed behavioural analysis of how social isolation affects male and female fly behaviour within a circular arena. We examine single-fly and same-sex groups of 10 flies within the arena and identify specific measures that are altered with social experience. In general, male and female behaviours are similarly affected by social deprivation. Lack of social interactions results in adult flies that exhibit less physical activity and are more socially avoidant of same-sex individuals. Furthermore, we report that presynaptic proteins in dopamine neurons are decreased in socially isolated fly brains. Surprisingly, we also show that presynaptic proteins generally are reduced across the entirety of the central brains of flies that lacked social experience, suggesting isolation leads to brain-wide changes in neural physiology and communication.

## 2. Material and Methods

### 2.1. Fly Strains and Husbandry

Canton-S *Drosophila melanogaster* flies (gift from RL Davis, Herbert Wertheim UF Scripps Institute for Biomedical Innovation & Technology, Jupiter, FL, USA) were used for sleep and fly arena experiments. They are an isogenic wildtype and have no transgenes present in their genomes, making them a common line used to study flies. The progeny from a cross between ~10 Canton-S females and ~7 Canton-S males were used for these experiments. *Drosophila melanogaster* transgenic strains used in this study are: TH-GAL4 (Bloomington *Drosophila* Stock Center, Bloomington, IN, USA, RRID: BDSC_8848) and UAS-Syt:eGFP (Bloomington *Drosophila* Stock Center, Bloomington, IN, USA, RRID: BDSC_6926).

### 2.2. Housing Conditions for Grouped and Isolated Flies

All flies were maintained on standard fly food and raised at 25 °C on a 12 h light:12 h dark cycle. All experiments were started/conducted on 5-day-old adult flies (i.e., 5 days after eclosion).

For the group-housed condition, parental flies were placed in a standard *Drosophila* vial containing fly food and given 24 h to lay eggs, after which the parents were removed. For the chronic social isolation condition, approximately 50 mL of standard fly food was melted then poured into a petri dish lined with parafilm and cooled. Once solidified, parental flies were placed onto the petri dish and given 24 h to lay eggs, after which parental flies were removed. *Drosophila* activity monitoring tubes (DAM tubes; TriKinetics Inc., Princeton, MA, USA) were used to punch out a section of fly food (~2 cm of food) containing one egg, then the end was sealed with parafilm. A cotton plug was used to seal the other end. One week later, after isolated flies had begun pupation, the old food was removed and replaced with new food to prevent the food from drying. Because flies were isolated throughout their life, isolated flies were not mated while group-housed flies were mated.

### 2.3. Sleep Assay

Sleep was assessed using the field standard *Drosophila* activity monitoring (DAM) system (TriKinetics Inc., Princeton, MA, USA). Food for the DAM sleep experiments was made according to standard practice (5% sucrose in a 2% agarose gel), added to each DAM tube, and the end wrapped with parafilm. Carbon dioxide was used to anesthetize and sex flies before loading into the DAM tubes. The open end of the tube was covered using a cotton plug. Fly housing condition and sex were positionally altered within the monitors such that each condition and sex had equal numbers of individuals along the monitor edges. The DAM monitors were incubated for 5 days at 25 °C with a 12:12 light:dark cycle.

Data were collected from the DAMSystem311 application 5 days after incubation. The first 24 h was discarded from data analysis (to allow recovery from CO_2_ anesthesia), and any dead flies at the end of testing were omitted from data analysis. The Sleep and Circadian Analysis MATLAB (v. 24.2.0.2740171) Program (S.C.A.M.P.) was used to analyze sleep data [49].

### 2.4. Fly Arena Assays

A FlyBowl arena [50] was 3D-printed and sanded with fine grit sandpaper to make smooth. A clear piece of acrylic was cut to the same size as the FlyBowl to serve as the lid. A box was made from white opaque acrylic with one open side (12 inches width × 12 inches depth × 9 inches height). A Raspberry Pi 4 circuit board (www.amazon.ca) was used to control a Raspberry Pi NoIR camera, mounted to the top of the cube to video record the flies at 30 frames per second for 5 min. A portable artist’s light table was used to achieve an even backlight of the arena for video recording. A blackout curtain was used to cover the open side of the box when video recording.

Group-housed flies were sexed 24–48 h prior to videoing under CO_2_ anesthesia. For single-fly arena assays, a single fly was aspirated into the arena without anesthesia. For social behaviour arena assays, 10 flies of a condition were all aspirated simultaneously then immediately transferred to the arena. Video 1 (5 min) was started immediately after placing the FlyBowl arena into the recording apparatus. Following acquisition of video 1, the arena was left in place for another 5 min (without recording). Then video 2 (5 min) was recorded 10–15 min after the flies were placed into the arena.

### 2.5. Video Tracking and Analysis

Arena videos were tracked and analyzed using CTrax (v. 0.5.18) and the MATLAB BehavioralMicroarray Toolbox (v. 0.2.24), according to usage documents (https://ctrax.sourceforge.net/bmat.html (accessed on 19 March 2026)) [51]. Errors were manually fixed for each video before data extraction. Thigmotaxis measures were obtained using the histogram properties function in the BehavioralMicroarray Toolbox (dist2wall parameter divided into 3 bins). Social proximity histogram data were obtained using the histogram properties function (dnose2ell parameter divided into 30 × 1 mm bins).

Three custom MATLAB scripts were written to extract specific data from these video tracking files, which have been made available as Supplemental Material files:

(1) DistanceCalc.m: to calculate the distances walked per fly (in mm);
(2) ApproachData.m: to identify approach bouts and to extract both average recording and approach bout specific behavioural parameters including social proximity to a focal fly’s nearest neighbour, approach velocity, speed, and number of approach bouts (used with per-frame stats MATLAB files). An approach bout was considered to have taken place if 2 flies close the distance between them (distance determined by the “dnose2ell” parameter) to at least less than 15 mm, although the vast majority of approach instances had minimum proximities less than 10 mm. Previous papers have used ~7.5 mm distance as a classification criterion for social interaction in control (group-housed) Canton-S flies [39]. We chose 15 mm based on our histogram data to be able to capture the major differences in grouped versus isolated flies, since isolated flies were more socially avoidant. Social proximity measures were calculated based on “dnose2ell” and approach velocity based on “veltoward_nose2ell” parameters.
(3) SpatialClustering.m: to extract social aggregation/clustering behaviour, including mean cluster number, mean flies per cluster, mean number of flies within a cluster, and mean number of flies not in a cluster (used with per-frame stats MATLAB files). Clustering was determined using a density-based data-clustering algorithm in MATLAB (dbscan function). Spatial clustering was assessed using 2 as the minimum number of points for a cluster and an epsilon value of 6. Epsilon value was determined empirically (by visualizing the classification of clusters and manual verification by an observer) using the GraphingSpatialClustering.m script. We have also included the GetEpsilon_for_SpatialClustering.m script in Supplemental Materials, which is used to theoretically calculate epsilon. However, we found the epsilon estimate returned via this theoretical method was much less conservative than cluster classification by manual observers and thus chose our epsilon value empirically.

### 2.6. Immunostaining

Immunostaining was conducted as previously described [52]. Five-day-old male or female fly brains were dissected in ice-cold Schneider’s insect medium (MilliporeSigma Canada Ltd., Oakville, ON, Canada, cat. #S0146). They were then placed in 1% PFA in Schneider’s insect medium at 4 °C overnight. Brains were then washed 3x in PAT3 (0.5% Triton X-100, 0.5% bovine serum albumin, in 1× PBS), after which blocking solution was added (3% normal goat serum in PAT3) for 1 h at room temperature on a nutator. Brains were then incubated with primary antibodies in a 3% normal goat serum in PAT3 solution for 3 h at room temperature, then 4 °C overnight (on a nutator throughout). Brains were washed 3× in PAT3, then incubated with the secondary antibody solution in 3% normal goat serum in PAT3 solution for 3 h at room temperature, then 4 °C for 5 days (on a nutator throughout). Brains were washed 3× in PAT3, rinsed quickly in 1× phosphate buffered saline, then quickly in dH_2_O before mounting between two glass coverslips separated by spacers. The primary antibodies used were: rabbit anti-GFP (1:1000, ThermoFisher Scientific, Mississauga, ON, Canada, A-11122, RRID: AB_221569) and mouse monoclonal anti-NC82 which labels Brp (1:50, Developmental Studies Hybridoma Bank, Iowa City, Iowa, USA, RRID: AB_2314866). The secondary antibodies used were: goat anti-rabbit Alexa FluorTM 488 (1:1000, ThermoFisher Scientific, Canada, A-11008, RRID: AB_143165) and goat anti-mouse Alexa FluorTM 633 (1:400, ThermoFisher Scientific, Canada, A-21050, RRID: AB_2535718).

Brains were imaged (anterior side closest to the objective lens) using a Leica Stellaris 5 inverted confocal microscope, using a water immersion 20× objective, with 488 nm and 633 nm laser excitation. Image stacks of brains were made with a 1μm step size. Images were then analyzed using ImageJ (v. 1.84p). Regions of interests (ROIs) were drawn across the whole central brain or specifically over the axons innervating regions of the mushroom body that were of interest. The mean fluorescence intensity values were calculated for structures in each of the hemispheres, then averaged to obtain one value per brain. Fluorescence measures were normalized and expressed as a percent of the group-housed condition for ease of data comparison.

### 2.7. Statistics

Statistics were calculated using GraphPad Prism 11 (v. 11.0.2). Data were assessed for normality using the Shapiro–Wilk test, then parametric or non-parametric tests were conducted as appropriate. The particular statistical test used for each experiment is identified in the figure legends. Sample sizes are indicated for each experiment within the figure itself. Significance was set at α = 0.05, and all tests were two-tailed. We did not correct for multiple comparisons in our dataset (with the exception of spatial location within the arena as fly location is mutually exclusive within the zones) to avoid a substantial and disproportionate risk of type 2 errors (false negatives) that would occur, whereas any possible type 1 errors (false positives) can be easily identified by experimental replication. We have replicated all the experiments within this manuscript (except for single-fly arena behaviours), and our results are overall consistent with those in the literature (see Discussion). All data used for analyses and figures in this manuscript are provided in the Supplementary Material file “Table S1. DataFile”.

## 3. Results

### 3.1. Social Isolation Reduces Sleep in Male but Not Female Drosophila

We socially isolated “wildtype” Canton-S flies by housing them in tubes used for the *Drosophila* activity monitoring (DAM) system (5 mm diameter × 65 mm length, TriKinetics) with standard food from embryos to 5–6-day-old young adult flies, while group-housed control flies were raised in standard *Drosophila* vials. We first assessed the effects of our isolation protocol on sleep, since chronically socially isolated male flies are known to sleep less than socially experienced male flies [12,15,16]. We replicated these findings here, as isolated male flies demonstrate reduced total night and day sleep durations (Figure 1A), as measured using the automated DAM system. Social isolation of female flies was previously shown not to reduce sleep [15]. Consistent with prior findings, we did not observe sleep loss in isolated female flies, although we demonstrated a slight but statistically significant increase in daytime sleep durations (Figure 1B). In general, our social isolation procedure replicates previous findings on how social deprivation differentially affects sleep in male and female *Drosophila*.

### 3.2. Social Isolation Reduces Single-Fly Exploratory Activity to a Novel Context

To observe fly behaviour within an arena, we used the previously established 3D printed circular arena (FlyBowl) [50] and overhead video recording captured using a Raspberry Pi circuit board and camera. A single fly was placed into the arena and 2 × 5 min videos were captured for behavioural analysis. The first video started immediately after flies were placed within the arena (video 1) followed by a second recording that started 10 min after they were placed within the arena (video 2; Figure 2A). Flies in the videos were tracked using Ctrax and the MATLAB BehavioralMicroarray toolkit [51], from which distance walked (mm), speed (mm/s), and spatial distribution within the arena (duration in different zones from arena wall) measures were extracted. In the 5 min following their introduction to the arena, isolated flies of both sexes explored the arena significantly less than group-housed controls, observable as reduced distance walked and reduced speed (Figure 2B,C), opposite to what was observed previously [45]. However, after 10 min of habituation to the arena, these group differences were no longer present (video 2; Figure S1A).

When examining the distance walked between video 1 and 2 within each condition, both group-housed males and females show a clear decrease in their exploratory behaviours within the novel arena, while isolated flies do not (although isolated male flies showed a trend towards a decrease; Figure S1B). We also quantified the time they spent in different parts of the arena within the 5 min videos but found no difference between group-housed or isolated flies (Figure 2C and Figure S1C). Thus, while social isolation appears to cause differences in a fly’s reaction to a novel arena, these initial differences either fade or are less pronounced after 10 min of habituation to the novel context.

### 3.3. Social Isolation Alters Same-Sex Group Locomotor Activity and Distribution Within an Arena

We then turned to examine how some social behaviours were altered with social deprivation in *Drosophila*. Using the same FlyBowl arena and procedure detailed above to observe single-fly behaviour, we instead aspirated 10 same-sex flies of the same housing condition into the arena without anesthesia (Figure 3A). We used a group of same-sex flies as we were interested in observing isolation effects on social behaviours independent of their effects on sexual motivations [47]. The 10 flies within the videos were individually tracked and behavioural measures were averaged for each video.

Similar to what we observed with single-fly behaviour in the arena, we again show a decrease in walking distance (mm) and speed (mm/s) in socially isolated flies of both sexes (Figure 3B). Interestingly, these behavioural changes appear to be more pronounced when observed in a group of flies compared to a single fly alone in an arena. The reduction in isolated male walking speed in these social interaction videos also persisted to video 2 (Figure S2A) while these differences were no longer apparent in single-fly arena video 2 (Figure S1A).

When 10 same-sex flies are placed in an arena, we start to observe changes in how socially experienced and isolated flies physically distribute within the FlyBowl arena (Figure 3C), which is different to what was observed of single-fly behaviour in the arena (Figure 2C). Male flies reared in chronic social isolation spend less time nearest the wall compared to group-housed flies and more time in the middle and central regions of the arena (Figure 3C). This general pattern of distribution also remained in video 2 (Figure S2C).

### 3.4. Social Isolation Increases Avoidance of Same-Sex Individuals

We focused on quantifying measures of social proximity, defined as the distance between nearest neighbour flies (measured from the nose of a focal fly to the closest point of its nearest neighbour; Figure 4A). Plotting a histogram of the amount of time flies spent in 1 mm bins of nearest neighbour proximity, the curve for both isolated males and females was shifted towards the right, meaning they spent less time within close social proximity to flies compared to group-housed flies (Figure 4B). If we average the nearest neighbour social proximity measure across the entirety of the recording, however, there was no observable difference in average social proximity (Figure 4C). But, if we specifically examine “approach bout” instances in which one or both flies are approaching their nearest neighbour, we observe a number of quantifiable differences. First is that upon each approach bout, isolated flies will not approach their nearest neighbour as closely as group-housed flies (Figure 4D). The number of approach bouts they perform is also significantly fewer than group-housed flies (Figure 4E), and the velocity of their approach towards their nearest neighbour is reduced (Figure 4F). Although the methodologies and specific measures we use here are different from those used previously, these results are consistent with the prior literature demonstrating isolated flies prefer to remain farther away from other conspecific flies [18,44,45,46].

Additionally, we used a density-based data clustering algorithm to analyze aggregation behaviour of the flies (Figure 4G). We find socially deprived flies form fewer clusters (Figure 4H). Singly housed female flies also form smaller clusters (Figure 4I) and overall fewer isolated flies are identified to be in a cluster across the recording compared to socially experienced flies (Figure 4J). This is also consistent with a previous paper that analyzed clustering [45]. Altogether, our approach bout and clustering measures indicate isolated flies are more socially avoidant than group-housed flies.

Most of these behavioural measures were no longer statistically different between grouped and isolated flies 10–15 min after the fly’s introduction to the arena (Figure S3). However, increased social distancing remained consistently affected in both socially isolated male and female flies (Figure S3A,C). An important caveat is that the lack of statistical differences in video 2 may be due to our low sample size (*n* = five videos). Thus, measures other than social proximity may be meaningfully and persistently different beyond the first 10 min within the arena.

### 3.5. Social Isolation Decreases Synaptic Proteins in Dopamine Neurons

Previously, isolated flies were shown to have decreased concentrations of dopamine in the brain compared to group-housed flies [16,40]. We expressed the synaptic vesicle marker, Synaptotagmin1—enhanced green fluorescent protein (Syt:eGFP) in the majority of dopamine neurons using the *TH-GAL4* driver line, via the *UAS-GAL4* binary expression system in *Drosophila* [53]. We previously demonstrated this method of assessing Syt:eGFP was a reliable marker for presynaptic proteins [54]. We assessed Syt:eGFP levels in dopamine neurons across the entirety of the central brain and found that both male and female isolated flies demonstrated reduced Syt:eGFP staining (Figure 5A,B), a result consistent with the prior report of lower brain dopamine concentrations upon social deprivation [16,40]. Previously, we have shown that increases in Syt:eGFP levels in specific dopamine neurons that innervate parts of the mushroom body, the PPL1-γ1pedc and PPL1-γ2α′1, were associated with olfactory learning and memory improvements [52,54]. We also specifically analyzed the Syt:eGFP levels from these neurons and observed significant decreases (Figure 5A,B), suggesting social isolation might also impair memory (aversive olfactory memory) [55], although this was not tested in this study.

Interestingly, we also found that the endogenous presynaptic protein, Bruchpilot (expressed in all neurons), was also significantly reduced in both isolated males and females (Figure 5C). Thus, social isolation appears to cause brain-wide deficits in synaptic proteins, likely affecting neural communication across multiple different neural circuit systems.

## 4. Discussion

We demonstrate that a wide variety of behaviours are altered in *Drosophila melanogaster* when they are socially isolated. Initially, the single-fly exploratory behaviour experiments within an arena were planned as a control experiment since we had not predicted exploratory activity to be altered with housing conditions. Thus, we were surprised to find that socially deprived flies display reduced walking distance and speed within a novel arena (Figure 2). Examining the behaviour of single flies from video 1 to video 2 (Figure S1B), we observe a clear statistical difference in the habituation of locomotor activity in grouped flies but not in isolated flies. Moreover, the average distance traveled and walking speed of group-housed flies in video 2 was similar to isolated flies in videos 1 and 2 (Figure 2 and Figure S1). These results suggest that isolated flies lack this initial elevated response to a novel arena. This could be explained by altered responses to, or recognition of, novelty, or by reduced arousal in the isolated flies. Further experiments will be required to test these possibilities.

In this study, we used small DAM tubes to isolate flies, while larger standard sized food vials were used to house grouped flies, which could potentially introduce confounds into our experiments. In previous studies on the behavioural effects of isolation on *Drosophila*, sleep, social proximity, and social clustering, we observe consistent behavioural effects of isolation whether the isolated flies are housed in large or the same-sized vials as grouped flies [12,33,44,45,46] or whether they are housed in smaller tubes [16,40]. Thus, the difference in the housing enclosure size is not likely to drive the behavioural effects of social deprivation observed here. One other important difference between our experimental groups is that while our group-housed flies were mated, our isolated flies were not. Mating status is also known to affect *Drosophila* female sleep profiles [56]. However, our sleep and arena-based social behaviour data are consistent with prior behavioural work on *Drosophila* social isolation, some of which tested mated group-housed and isolated flies [12,33,46]. Because the behavioural differences observed are consistent whether isolation occurs in a standard vial or smaller tube (e.g., DAM tube) and regardless of differences in mating status, it suggests these behavioural abnormalities arise primarily from the lack of social interactions itself.

Social isolation is known to result in abnormal social behaviours in many different species, including in *Drosophila.* Socially isolated *Drosophila* are known to display abnormal mating behaviours [47], higher levels of aggression [28], and decreased proximity to other individuals [18,40,44,45,46]. Here, we used video recordings to assess social behaviour within a group of 10 same-sex flies. We replicate the prior findings that used the social space assay [18,46] in that isolated flies maintain farther distances from each other (in video 1 and 2; Figure 4D and Figure S3C) compared with group-housed individuals. They also tend to approach each other less and are less likely to form into spatial clusters (Figure 4E–J). Taken together, these social behaviour measures are consistent with the idea that socially deprived individuals are more socially avoidant than control group-housed flies.

Previous studies have assessed a variety of measures of social behaviours in genetically variable strains of *Drosophila*, demonstrating that some aspects of sociality are heritable [39,57,58,59]. When examining the relationship between social proximity and locomotor activity in different genetically variable strains of *Drosophila melanogaster*, there does not appear to be a consistent relationship between these two behaviours [57,58,59]. One prior study on genetically variable strains of *Drosophila melanogaster* found a negative correlation between social proximity distances and time in the centre of the arena [57], which would suggest these measures might share a mechanistic relationship. However, we find the opposite changes when manipulating social housing conditions (Figure 3D and Figure 4D). Interestingly, when we assayed single-fly videos, isolated flies did not show an increased bias towards the middle or centre parts of the arena compared to group-housed flies (Figure 2C and Figure S1C). Thus, we speculate that the spatial distribution differences of isolated flies towards more central regions of the arena when in groups of 10 may arise from their tendency for social avoidance, rather than from a true preference for the central regions of an arena. Of note is that for our measures of fly distribution within our circular arena, due to the way in which we designate different areas of the arena for analyses (Figure 2C, Figure 3D, Figures S1C and S2C), there are differences in area sizes for each region in a 5:3:1 ratio for wall:middle:centre regions. If we correct our data for these area differences, flies will typically spend most of their time in the wall or middle regions, but consistently spend much less time in the centre, suggesting they may innately display centrophobic behaviour.

Socially deprived *Drosophila* were previously identified to have reduced concentrations of dopamine in the brain [16,40]. Moreover, genetic manipulations of the dopaminergic system or dopamine receptor mutants reveal that dopamine appears to promote social proximity in *Drosophila* as assessed using the social space assay [46,48]. Our findings that synaptic proteins are decreased in the dopaminergic neurons of isolated flies (Figure 5A,B), and that isolated flies are more socially avoidant (Figure 4 and Figure S3), are consistent with these previously reported data. While dopamine neuron activity is positively correlated with physical movement in flies, their artificial activation does not cause an increase in locomotor activity [60]. However, the areas from which we measured Syt:eGFP levels in dopamine neurons are known to be important for learning and memory [38,55]. Levels of Syt:eGFP in these dopamine neurons are also positively associated with aversive olfactory memory performance [52,54], suggesting that isolated flies would exhibit impairments in this memory task. Social deprivation in rodents and in humans is also repeatedly connected to poor learning and memory [5,23,26,61,62,63].

Interestingly, however, we find that these decreases in synaptic proteins extend to the whole central brain (Figure 5C). Thus, isolation may cause decreased levels of synaptic proteins generally, and these effects may not be specific to the dopaminergic system. Although our microscope images are qualitatively consistent with this interpretation, we cannot exclude the possibility that the differences in whole-brain measures of synaptic proteins may be largely driven by changes in a limited number of brain structures. These results suggest that neural communication between neurons is generally depressed across the brain; however, this will have to be confirmed in future studies. Moreover, the brain-wide synaptic effects observed are superficially consistent with the observations that a wide variety of both non-social and social behaviours are affected by isolation.

We observe a sex difference in isolation effects on sleep, where isolated males exhibit reductions in sleep (consistent with previously published studies [12,15,16]), while female flies do not or perhaps increase their sleep (Figure 1A,B). We have replicated this social isolation and sleep experiment as part of a separate study, with the replication demonstrating no effect of isolation on sleep in females, consistent with previously published data [15]. Taken together, we can state that social deprivation reduces sleep in male flies but not in female flies. The mechanisms leading to this sex difference in sleep are unclear; however, there are known sex differences in fly sleep, some of which are regulated by juvenile hormones [64]. In contrast to the effects of isolation on sleep, we were rather surprised to find that arena-based behaviours were not different between male and female flies. It is possible that other subtle behaviours not measured here may demonstrate sex differences. On the other hand, there does seem to be a large difference in the magnitude of Brp decrease observed in female flies compared to male flies (Figure 5C). However, we have performed a separate set of experiments to assess whole-brain levels of a different presynaptic protein, Synaptotagmin1 as part of a different study, and found no consistent differences in the magnitude of changes between the sexes. For these reasons, the large magnitude of decrease in Brp staining in isolated females compared to males may be due to experimental variation rather than a sex difference. Future experiments are planned to investigate whether there is a meaningful sex difference in the Brp presynaptic protein levels of isolated brains.

## 5. Conclusions

*Drosophila* experiencing a lack of social interactions display a surprising number of behavioural changes observable within an arena, demonstrating the importance of social experience for a normal behavioural profile. We outline several key behavioural parameters that are consistently altered upon social isolation, which can serve as reliable indicators of behavioural changes associated with social deprivation in *Drosophila*. Moreover, social isolation leads to measurable decreases in presynaptic proteins in dopamine neurons and across the entirety of the brain, indicating systemic brain alterations occur in the absence of social experience.

## Figures and Tables

**Figure 1:**
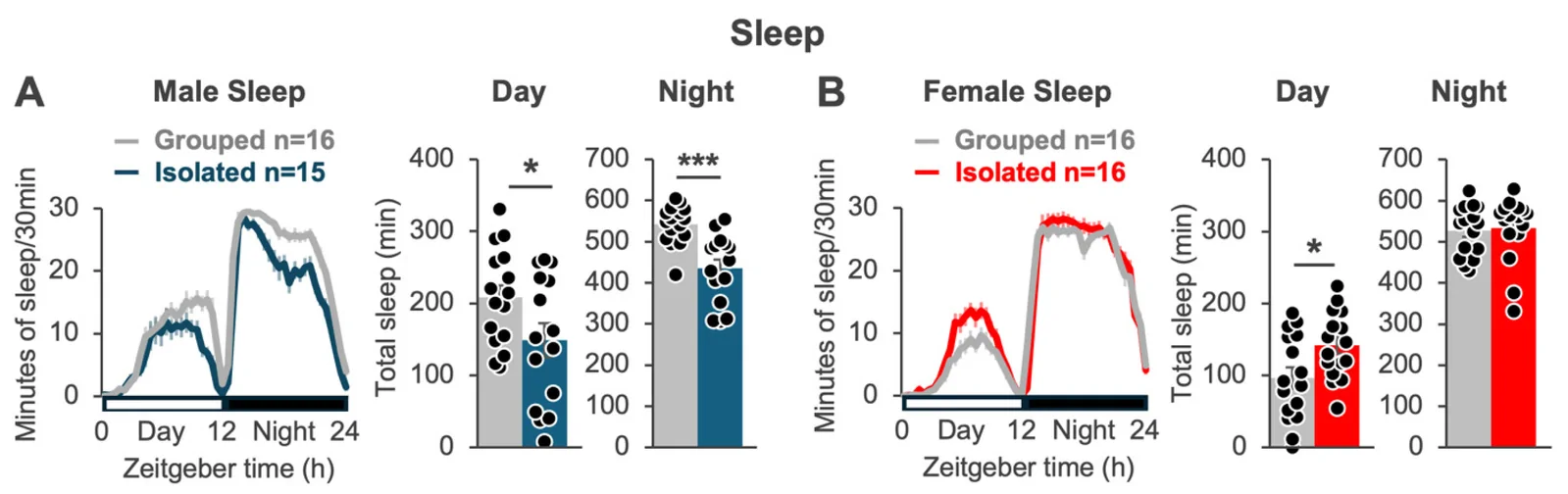
Social isolation affects sleep behaviour differently in male and female *Drosophila.* Average duration of sleep in male and female flies across 4 days and nights calculated from activity levels measured by the automated *Drosophila* activity monitoring (DAM) system. (**A**) Both day and night sleep durations are significantly reduced in isolated male flies compared to group-housed male flies. Unpaired *t*-test (day t = 2.074, df = 29, *p* = 0.0471; night t = 4.413, df = 29, *p* = 0.0001). (**B**) Isolated female flies exhibited an increase in day-time sleep durations but no difference in night sleep durations compared to group-housed female flies. Unpaired *t*-test (day t = 2.41, df = 30, *p* = 0.022). All graphs mean ± SEM, * *p* < 0.05, *** *p* < 0.001.

**Figure 2:**
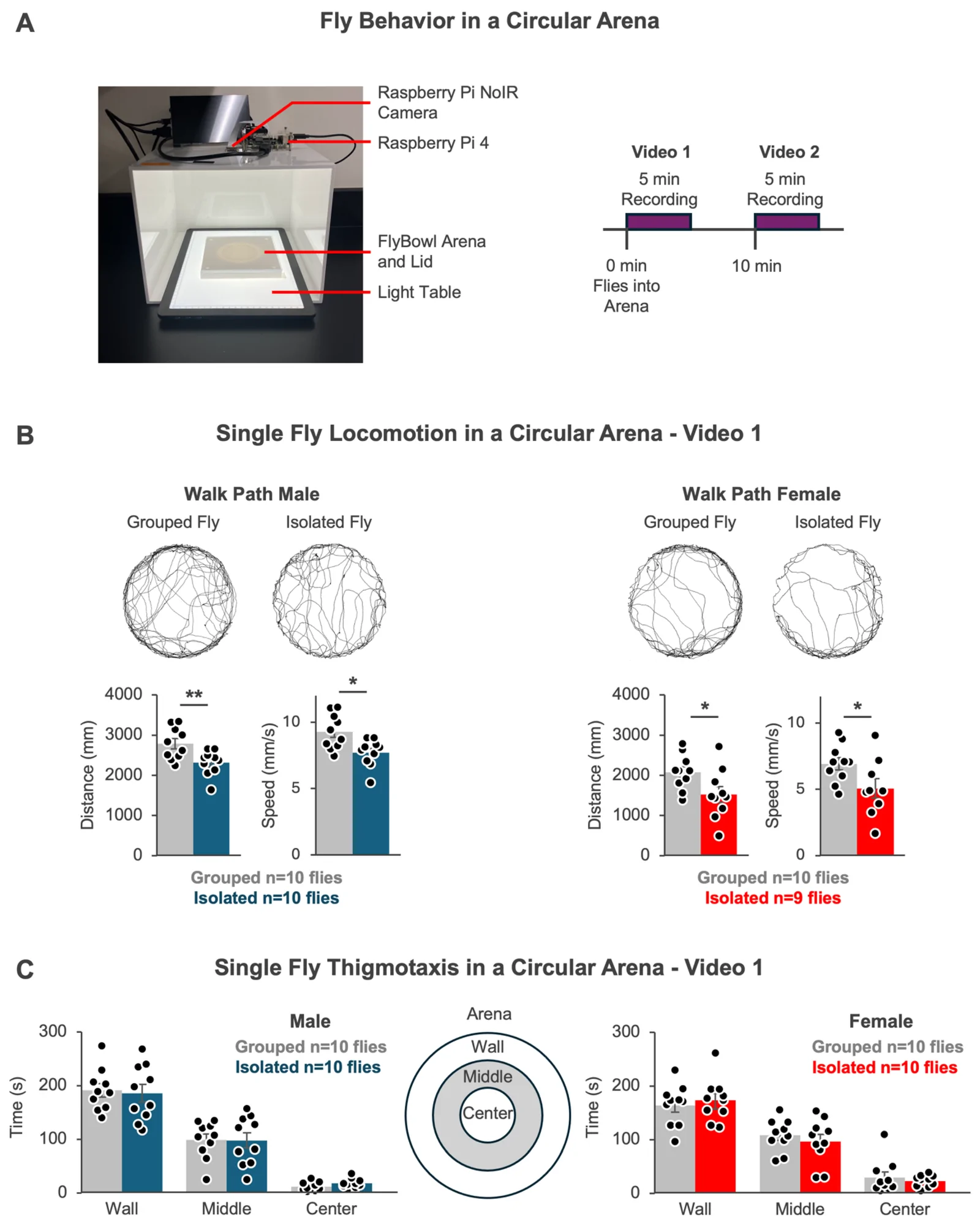
Social isolation reduces locomotion of single flies in a circular arena. Single flies were placed into the FlyBowl arena and tracked using cTrax and the BehavioralMicroarray MATLAB toolkit. (**A**) Behavioural video recording setup and timing of data collection. (**B**) Isolated male and female flies displayed decreased walk distances and speeds in video 1. Unpaired *t*-tests (males: distance t = 2.952, df = 18, *p* = 0.0085; speed t = 2.955, df = 18, *p* = 0.0085; females: distance t = 2.312, df = 18, *p* = 0.033; speed t = 2.196, df = 17, *p* = 0.042). (**C**) Different housing conditions of flies do not alter the spatial distribution of single flies within an arena in video 1. All graphs mean ± SEM, * *p* < 0.05, ** *p* < 0.01.

**Figure 3:**
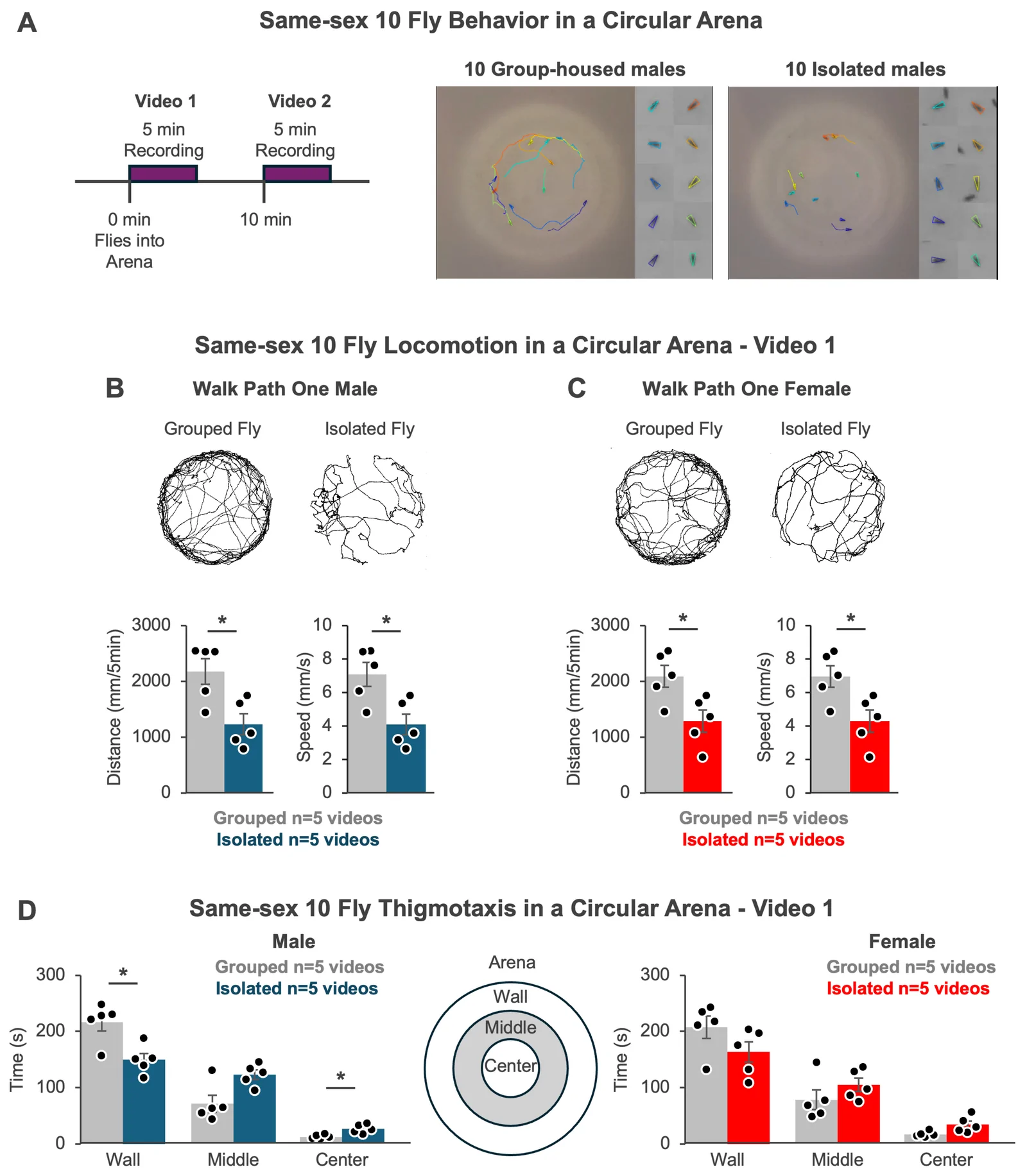
Isolated flies demonstrate reduced locomotion and spatial distributions when in groups of 10 same-sex flies. Ten same-sex flies of the same housing condition were placed into the FlyBowl arena, then tracked using cTrax and the BehavioralMicroarray MATLAB toolkit. (**A**) Timing of behavioural data collection and example of tracking from our video recordings. (**B**) Isolated male flies display reduced distances walked and speed within the FlyBowl arena compared to group-housed male flies in video 1. Unpaired *t*-test (distance t = 3.182, df = 8, *p* = 0.013; speed t = 3.129, df = 8, *p* = 0.014). (**C**) Isolated female flies display reduced distances walked and speed in groups of 10 compared to group-housed female flies in video 1. Unpaired *t*-test (distance t = 2.894, df = 8, *p* = 0.021; speed t = 2.886, df = 8, *p* = 0.0203). (**D**) The spatial distribution of group-housed and isolated flies within the FlyBowl arena differs when in 10 same-sex fly groups, with isolated flies preferring centre parts of the arena compared to the wall. Unpaired *t*-tests with Bonferroni correction (males: wall t = 3.462, df = 8, adjusted *p* = 0.026; centre t = 3.506, df = 8, adjusted *p* = 0.024). All graphs mean ± SEM, * *p* < 0.05.

**Figure 4:**
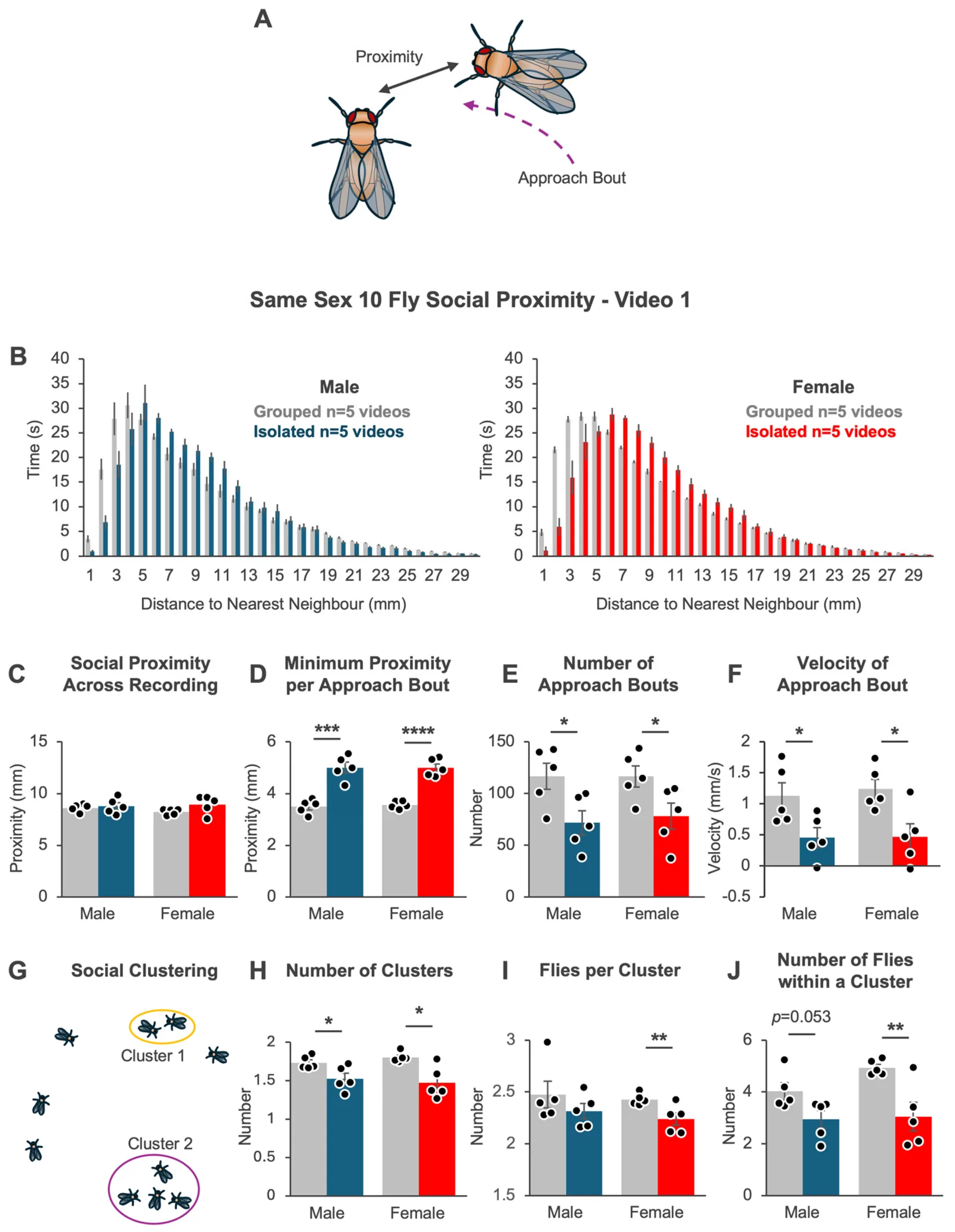
Isolated flies are more socially avoidant than group-housed flies. Measures associated with social proximity and social clustering during video 1. (**A**) Illustration of proximity and approach behaviour. (**B**) Time spent in 1 mm bins of proximity to a fly’s nearest neighbour. (**C**) Isolated flies do not differ in their average social proximity across video 1. (**D**) Upon an approach bout, group-housed flies approach each other more closely than isolated flies. Unpaired *t*-test (males: t = 6.179, df = 8, *p* = 0.0003; females: t = 8.709, df = 8, *p* < 0.0001). (**E**) Isolated flies also perform fewer approach bouts than group-housed flies. Unpaired *t*-test (males: t = 2.604, df = 8, *p* = 0.0314; females: t = 2.369, df = 8, *p* = 0.0453). (**F**) Isolated flies also demonstrate reduced velocity per approach bout. Unpaired *t*-test (males: t = 2.511, df = 8, *p* = 0.0363; females: t = 2.973, df = 8, *p* = 0.0178). (**G**) Example of clustering of flies within an arena. (**H**) Isolated male and female flies form fewer clusters than group-housed flies. Unpaired *t*-test (males: t = 2.563, df = 8, *p* = 0.0335; females: t = 2.873, df = 8, *p* = 0.0207). (**I**) Group-housed females form larger clusters than isolated females. Unpaired *t*-test (females: t = 3.430, df = 8, *p* = 0.009). (**J**) The number of flies within a cluster across video 1 was significantly decreased in female isolated flies. Unpaired *t*-test (females: t = 3.384, df = 8, *p* = 0.0096). All graphs mean ± SEM, * *p* < 0.05, ** *p* < 0.01, *** *p* < 0.001, **** *p* < 0.0001.

**Figure 5:**
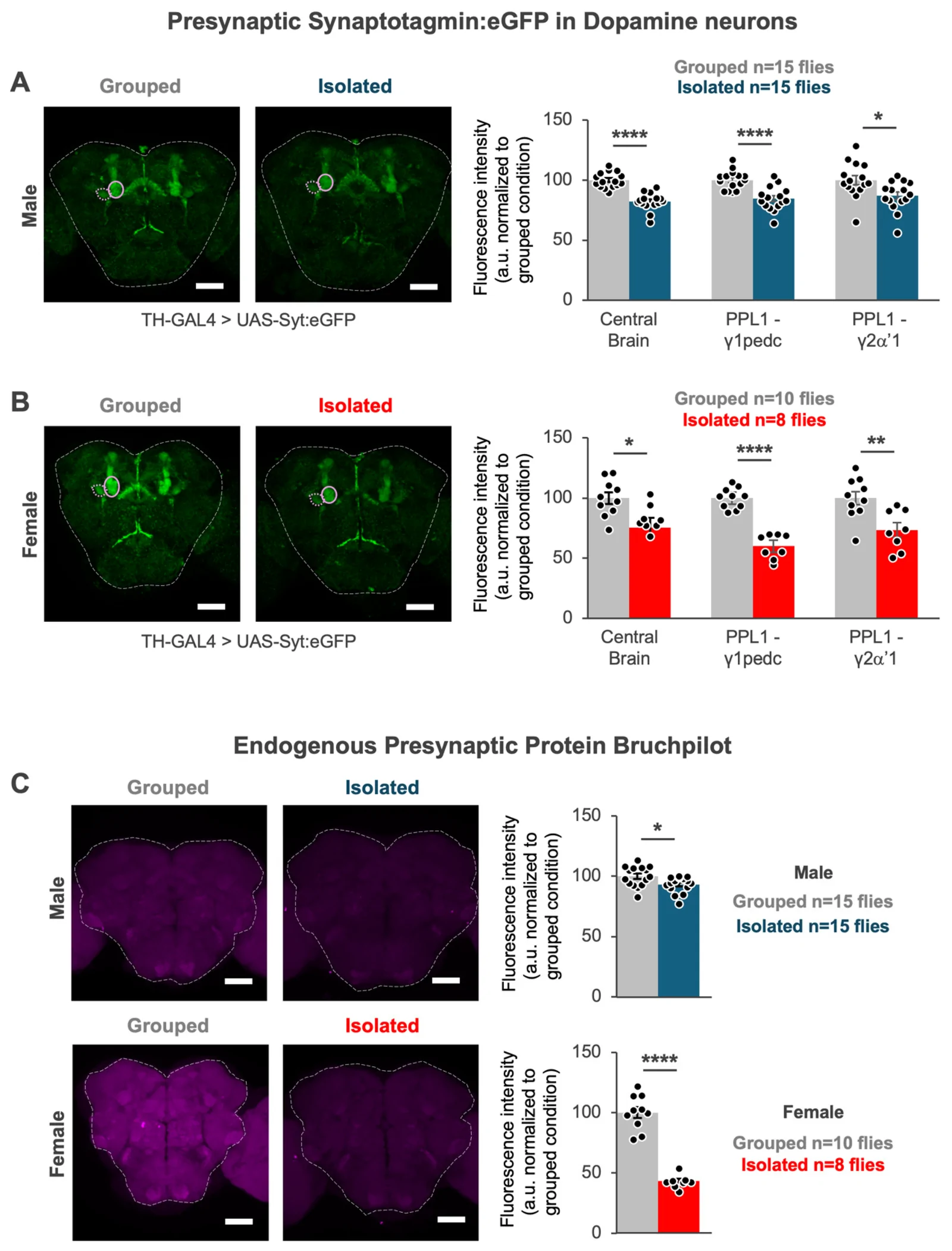
Synaptic proteins in dopamine neurons and across the whole brain are decreased in socially isolated flies. (**A**) Socially deprived male flies have reduced levels of Syt:eGFP in dopamine neurons across the central brain and in at least two areas where they innervate the mushroom body structure. Unpaired *t*-test (central brain: t = 7.130, df = 28, *p* < 0.0001; PPL1- γ1pedc: t = 4.701, df = 28, *p* < 0.0001; PPL1-γ2α′1: t = 2.609, df = 28, *p* = 0.0144). (**B**) Socially isolated female flies also show reduced levels of Syt:eGFP in dopamine neurons across the central brain and in at least two areas where they innervate the mushroom body structure. Unpaired *t*-test (central brain: t = 2.671, df = 16, *p* = 0.0167; PPL1- γ1pedc: t = 8.981, df = 16, *p* < 0.0001; PPL1-γ2α′1: t = 3.276, df = 16, *p* = 0.0048). (**C**) Endogenous levels of presynaptic neuronal marker Bruchpilot are also significantly reduced across the central brain in both isolated male and female brains. Mann–Whitney test (males: U = 63, *p* = 0.0408) or unpaired *t*-test (females: t = 10.63, df = 16, *p* < 0.0001). White dashed line outlines the central brain. Pink dotted line outlines the dopamine neuron PPL1- γ1pedc axons; pink solid line outlines the dopamine neuron PPL1-γ2α′1 axons. All graphs mean ± SEM, * *p* < 0.05, ** *p* < 0.01, **** *p* < 0.0001.

## Data Availability

The raw data supporting the conclusions of this article are available in Supplementary Materials Table S1: DataFile.

## Supplementary Materials

The following supporting information can be downloaded at: https://www.mdpi.com/article/10.3390/biology15191721/s1, Figure S1: Locomotion of single flies within an arena after 10 min of habituation; Figure S2: Locomotion of 10 same sex flies within an arena after 10 min of habituation; Figure S3: Measures of social proximity in group-housed and isolated flies after 10 min of habituation; Table S1: DataFile. MATLAB files used to obtain specific behavioural data as described in the Methods section: ApproachData.m, DistanceCalc.m, GetEpsilon_for_spatialClustering.m, GraphingSpatialClustering.m, SpatialClustering.m.

## References

[1] Coyle C.E., Dugan E. (2012). Social Isolation, Loneliness and Health Among Older Adults. J. Aging Health.

[2] Lee E.E., Depp C., Palmer B.W., Glorioso D., Daly R., Liu J., Tu X.M., Kim H.-C., Tarr P., Yamada Y. (2019). High Prevalence and Adverse Health Effects of Loneliness in Community-Dwelling Adults across the Lifespan: Role of Wisdom as a Protective Factor. Int. Psychogeriatr..

[3] Cacioppo J.T., Hawkley L.C., Thisted R.A. (2010). Perceived Social Isolation Makes Me Sad: 5-Year Cross-Lagged Analyses of Loneliness and Depressive Symptomatology in the Chicago Health, Aging, and Social Relations Study. Psychol. Aging.

[4] Barth J., Schneider S., von Känel R. (2010). Lack of Social Support in the Etiology and the Prognosis of Coronary Heart Disease: A Systematic Review and Meta-Analysis. Psychosom. Med..

[5] Wilson R.S., Krueger K.R., Arnold S.E., Schneider J.A., Kelly J.F., Barnes L.L., Tang Y., Bennett D.A. (2007). Loneliness and Risk of Alzheimer Disease. Arch. Gen. Psychiatry.

[6] Buecker S., Horstmann K.T. (2021). Loneliness and Social Isolation During the COVID-19 Pandemic. Eur. Psychol..

[7] Brandt L., Liu S., Heim C., Heinz A. (2022). The Effects of Social Isolation Stress and Discrimination on Mental Health. Transl. Psychiatry.

[8] York Centre for Reviews and Dissemination (2014). Interventions for Loneliness and Social Isolation.

[9] Xiong Y., Hong H., Liu C., Zhang Y.Q. (2023). Social Isolation and the Brain: Effects and Mechanisms. Mol. Psychiatry.

[10] Cacioppo J.T., Hawkley L.C. (2003). Social Isolation and Health, with an Emphasis on Underlying Mechanisms. Perspect. Biol. Med..

[11] Kaushal N., Nair D., Gozal D., Ramesh V. (2012). Socially Isolated Mice Exhibit a Blunted Homeostatic Sleep Response to Acute Sleep Deprivation Compared to Socially Paired Mice. Brain Res..

[12] Li W., Wang Z., Syed S., Lyu C., Lincoln S., O’Neil J., Nguyen A.D., Feng I., Young M.W. (2021). Chronic Social Isolation Signals Starvation and Reduces Sleep in *Drosophila*. Nature.

[13] Kurina L.M., Knutson K.L., Hawkley L.C., Cacioppo J.T., Lauderdale D.S., Ober C. (2011). Loneliness Is Associated with Sleep Fragmentation in a Communal Society. Sleep.

[14] Pilcher J.J., Dorsey L.L., Galloway S.M., Erikson D.N. (2022). Social Isolation and Sleep: Manifestation During COVID-19 Quarantines. Front. Psychol..

[15] Zhao H., Jiang X., Ma M., Xing L., Ji X., Pan Y. (2024). A Neural Pathway for Social Modulation of Spontaneous Locomotor Activity (SoMo-SLA) in *Drosophila*. Proc. Natl. Acad. Sci. USA.

[16] Ganguly-Fitzgerald I., Donlea J., Shaw P.J. (2006). Waking Experience Affects Sleep Need in *Drosophila*. Science.

[17] Arzate-Mejía R.G., Lottenbach Z., Schindler V., Jawaid A., Mansuy I.M. (2020). Long-Term Impact of Social Isolation and Molecular Underpinnings. Front. Genet..

[18] Simon A.F., Chou M.-T., Salazar E.D., Nicholson T., Saini N., Metchev S., Krantz D.E. (2012). A Simple Assay to Study Social Behavior in *Drosophila*: Measurement of Social Space within a Group. Genes Brain Behav..

[19] Vora A., Nguyen A.D., Spicer C., Li W. (2022). The Impact of Social Isolation on Health and Behavior in *Drosophila melanogaster* and Beyond. Brain Sci. Adv..

[20] Killgore W.D.S., Cloonan S.A., Taylor E.C., Anlap I., Dailey N.S. (2021). Increasing Aggression during the COVID-19 Lockdowns. J. Affect. Disord. Rep..

[21] Zelikowsky M., Hui M., Karigo T., Choe A., Yang B., Blanco M.R., Beadle K., Gradinaru V., Deverman B.E., Anderson D.J. (2018). The Neuropeptide Tac2 Controls a Distributed Brain State Induced by Chronic Social Isolation Stress. Cell.

[22] Takahashi A. (2022). The Role of Social Isolation Stress in Escalated Aggression in Rodent Models. Neurosci. Res..

[23] Segel-Karpas D., Ayalon L. (2020). Loneliness and Hostility in Older Adults: A Cross-Lagged Model. Psychol. Aging.

[24] Eddison M. (2021). A Genetic Screen for *Drosophila* Social Isolation Mutants and Analysis of Sex Pistol. Sci. Rep..

[25] Chiu H., Hoopfer E.D., Coughlan M.L., Pavlou H.J., Goodwin S.F., Anderson D.J. (2021). A Circuit Logic for Sexually Shared and Dimorphic Aggressive Behaviors in *Drosophila*. Cell.

[26] Kawanabe S., Mori M., Harada H., Murata Y., Ohe K., Enjoji M. (2023). Upregulations of A1 Adrenergic Receptors and Noradrenaline Synthases in the Medial Prefrontal Cortex Are Associated with Emotional and Cognitive Dysregulation Induced by Post-Weaning Social Isolation in Male Rats. Neurosci. Lett..

[27] Asahina K., Watanabe K., Duistermars B.J., Hoopfer E., González C.R., Eyjólfsdóttir E.A., Perona P., Anderson D.J. (2014). Tachykinin-Expressing Neurons Control Male-Specific Aggressive Arousal in *Drosophila*. Cell.

[28] Agrawal P., Kao D., Chung P., Looger L.L. (2020). The Neuropeptide Drosulfakinin Regulates Social Isolation-Induced Aggression in *Drosophila*. J. Exp. Biol..

[29] Leech T., Sait S.M., Bretman A. (2017). Sex-Specific Effects of Social Isolation on Ageing in *Drosophila melanogaster*. J. Insect Physiol..

[30] Yadav R.S.P., Ansari F., Bera N., Kent C., Agrawal P. (2024). Lessons from Lonely Flies: Molecular and Neuronal Mechanisms Underlying Social Isolation. Neurosci. Biobehav. Rev..

[31] Sokolowski M.B. (2010). Social Interactions in “Simple” Model Systems. Neuron.

[32] Mery F., Varela S.A.M., Danchin É., Blanchet S., Parejo D., Coolen I., Wagner R.H. (2009). Public Versus Personal Information for Mate Copying in an Invertebrate. Curr. Biol..

[33] Jiang L., Cheng Y., Gao S., Zhong Y., Ma C., Wang T., Zhu Y. (2020). Emergence of Social Cluster by Collective Pairwise Encounters in *Drosophila*. Elife.

[34] Ramdya P., Lichocki P., Cruchet S., Frisch L., Tse W., Floreano D., Benton R. (2015). Mechanosensory Interactions Drive Collective Behaviour in *Drosophila*. Nature.

[35] Durisko Z., Kemp R., Mubasher R., Dukas R. (2014). Dynamics of Social Behavior in Fruit Fly Larvae. PLoS ONE.

[36] Dombrovski M., Kim A., Poussard L., Vaccari A., Acton S., Spillman E., Condron B., Yuan Q. (2019). A Plastic Visual Pathway Regulates Cooperative Behavior in *Drosophila* Larvae. Curr. Biol..

[37] Dombrovski M., Poussard L., Moalem K., Kmecova L., Hogan N., Schott E., Vaccari A., Acton S., Condron B. (2017). Cooperative Behavior Emerges among *Drosophila* Larvae. Curr. Biol..

[38] Dwijesha A.S., Eswaran A., Berry J.A., Phan A. (2024). Diverse Memory Paradigms in *Drosophila* Reveal Diverse Neural Mechanisms. Learn. Mem..

[39] Rooke R., Rasool A., Schneider J., Levine J.D. (2020). *Drosophila melanogaster* Behaviour Changes in Different Social Environments Based on Group Size and Density. Commun. Biol..

[40] Petrović M., Medija M., Martinović L.S., Meštrović A., Waldowski R.A., Vujnović A.F. (2026). Pharmacological Rescue of Isolation-Induced Social and Neurochemical Deficits in *Drosophila melanogaster*. Sci. Rep..

[41] Scheffer L.K., Xu C.S., Januszewski M., Lu Z., Takemura S., Hayworth K.J., Huang G.B., Shinomiya K., Maitlin-Shepard J., Berg S. (2020). A Connectome and Analysis of the Adult *Drosophila* Central Brain. Elife.

[42] Bates A.S., Phelps J.S., Kim M., Yang H.H., Matsliah A., Ajabi Z., Perlman E., Delgado K.M., Osman M.A.M., Salmon C.K. (2026). Distributed Control Circuits across a Brain-and-Cord Connectome. Nature.

[43] Venken K.J.T., Simpson J.H., Bellen H.J. (2011). Genetic Manipulation of Genes and Cells in the Nervous System of the Fruit Fly. Neuron.

[44] Sun Y., Qiu R., Li X., Cheng Y., Gao S., Kong F., Liu L., Zhu Y. (2020). Social Attraction in *Drosophila* Is Regulated by the Mushroom Body and Serotonergic System. Nat. Commun..

[45] Bentzur A., Ben-Shaanan S., Benichou J.I.C., Costi E., Levi M., Ilany A., Shohat-Ophir G. (2021). Early Life Experience Shapes Male Behavior and Social Networks in *Drosophila*. Curr. Biol..

[46] Xie T., Ho M.C.W., Liu Q., Horiuchi W., Lin C.-C., Task D., Luan H., White B.H., Potter C.J., Wu M.N. (2018). A Genetic Toolkit for Dissecting Dopamine Circuit Function in *Drosophila*. Cell Rep..

[47] Sethi S., Lin H.-H., Shepherd A.K., Volkan P.C., Su C.-Y., Wang J.W. (2019). Social Context Enhances Hormonal Modulation of Pheromone Detection in *Drosophila*. Curr. Biol..

[48] Fernandez R.W., Akinleye A.A., Nurilov M., Feliciano O., Lollar M., Aijuri R.R., O’Donnell J.M., Simon A.F. (2017). Modulation of Social Space by Dopamine in *Drosophila melanogaster*, but No Effect on the Avoidance of the *Drosophila* Stress Odorant. Biol. Lett..

[49] Donelson N., Kim E.Z., Slawson J.B., Vecsey C.G., Huber R., Griffith L.C. (2012). High-Resolution Positional Tracking for Long-Term Analysis of *Drosophila* Sleep and Locomotion Using the “Tracker” Program. PLoS ONE.

[50] Simon J.C., Dickinson M.H. (2010). A New Chamber for Studying the Behavior of *Drosophila*. PLoS ONE.

[51] Branson K., Robie A.A., Bender J., Perona P., Dickinson M.H. (2009). High-Throughput Ethomics in Large Groups of *Drosophila*. Nat. Methods.

[52] Pimenov I., MacMullen C.M., Ezeh C., Sai Dwijesha A., David J., Eswaran A., Davis R.L., Phan A. (2026). Dopamine Neuron Specific RNA-Sequencing Reveals Neprilysin 1 Acts Downstream of the Cohesin Complex to Suppress Learning. Commun. Biol..

[53] Brand A.H., Perrimon N. (1993). Targeted Gene Expression as a Means of Altering Cell Fates and Generating Dominant Phenotypes. Development.

[54] Phan A., Thomas C.I., Chakraborty M., Berry J.A., Kamasawa N., Davis R.L. (2019). Stromalin Constrains Memory Acquisition by Developmentally Limiting Synaptic Vesicle Pool Size. Neuron.

[55] Davis R.L. (2023). Learning and Memory Using *Drosophila melanogaster*: A Focus on Advances Made in the Fifth Decade of Research. Genetics.

[56] Bailly T.P.M., Lamers S.J.C., Sarma A., Jansen A.C.M., Freerks K., van Dijk M., Etienne R.S., Wertheim B., Billeter J.-C. (2026). Sociability Is a Multidimensional Trait in *Drosophila melanogaster*. Behav. Genet..

[57] Anderson B.B., Scott A., Dukas R. (2017). Indirect Genetic Effects on the Sociability of Several Group Members. Anim. Behav..

[58] Scott A.M., Dworkin I., Dukas R. (2018). Sociability in Fruit Flies: Genetic Variation, Heritability and Plasticity. Behav. Genet..

[59] Berry J.A., Cervantes-Sandoval I., Chakraborty M., Davis R.L. (2015). Sleep Facilitates Memory by Blocking Dopamine Neuron-Mediated Forgetting. Cell.

[60] Kang J.W., Oremus M. (2023). Examining the Combined Effects of Social Isolation and Loneliness on Memory: A Systematic Review. Arch. Gerontol. Geriatr..

[61] Benfato I.D., Quintanilha A.C.S., Henrique J.S., Souza M.A., Rosário B.d.A., Beserra Filho J.I.A., Santos R.L.O., Ribeiro A.M., Le Sueur Maluf L., de Oliveira C.A.M. (2022). Effects of Long-Term Social Isolation on Central, Behavioural and Metabolic Parameters in Middle-Aged Mice. Behav. Brain Res..

[62] Wang B., Wu Q., Lei L., Sun H., Michael N., Zhang X., Wang Y., Zhang Y., Ge B., Wu X. (2019). Long-Term Social Isolation Inhibits Autophagy Activation, Induces Postsynaptic Dysfunctions and Impairs Spatial Memory. Exp. Neurol..

[63] Ibi D., Takuma K., Koike H., Mizoguchi H., Tsuritani K., Kuwahara Y., Kamei H., Nagai T., Yoneda Y., Nabeshima T. (2008). Social Isolation Rearing-Induced Impairment of the Hippocampal Neurogenesis Is Associated with Deficits in Spatial Memory and Emotion-Related Behaviors in Juvenile Mice. J. Neurochem..

[64] Wu B., Ma L., Zhang E., Du J., Liu S., Price J., Li S., Zhao Z. (2018). Sexual Dimorphism of Sleep Regulated by Juvenile Hormone Signaling in *Drosophila*. PLoS Genet..

