# Supplementary material for: Social Isolation Causes Behavioural Abnormalities and Reduces Synaptic Proteins in the Brains of Drosophila
Source: Biology (Basel). 2026 Sep 30;15(19):1721. doi: 10.3390/biology15191721 (PMC13663386; doi:10.3390/biology15191721)
Supplement: Supplementary file 1 [file biology-15-01721-s001.zip › Supplementary Figures Social isolation and Social Prox.pdf]

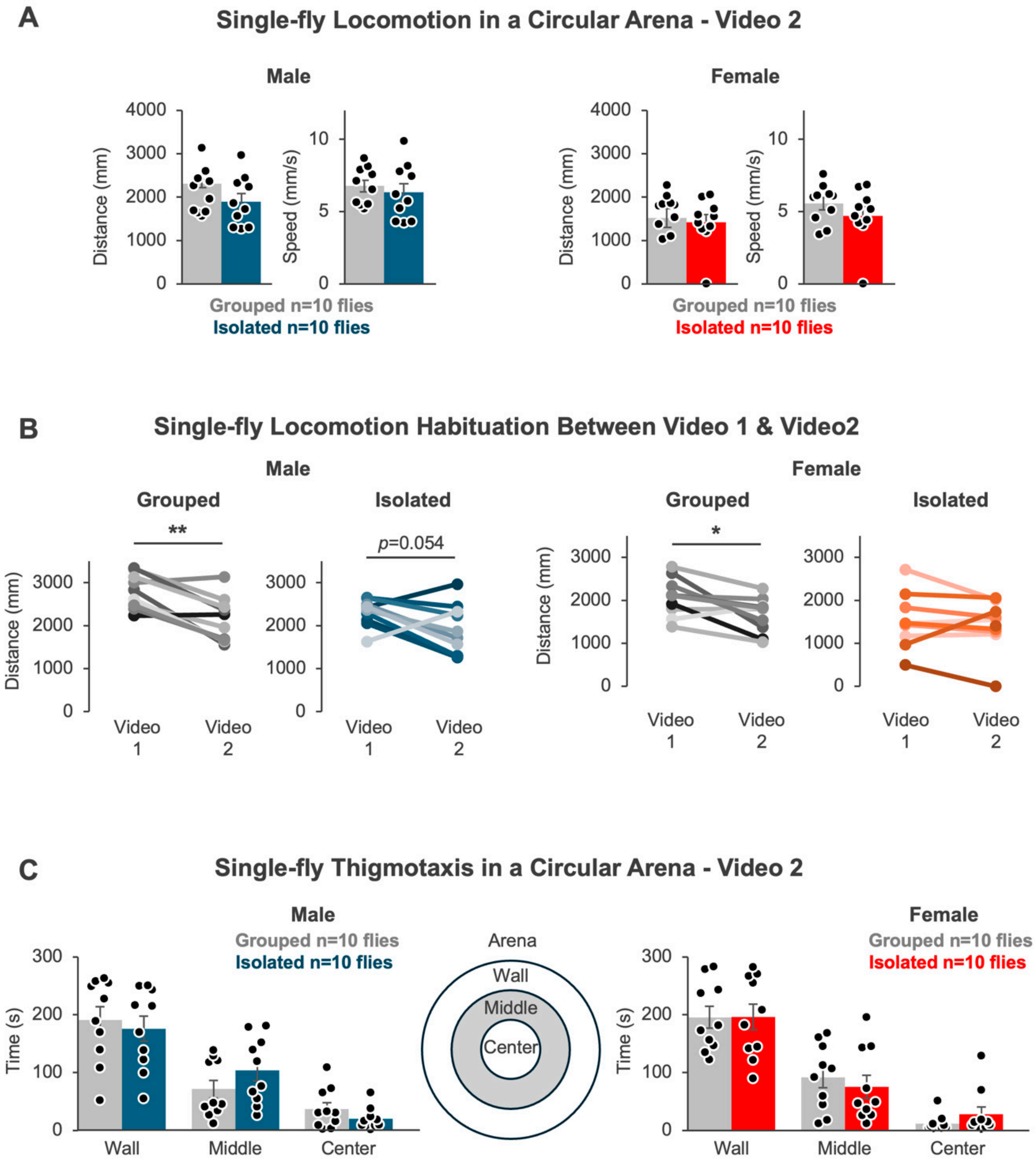

Figure S1. Locomotion of single flies within an arena after 10min of habituation. **(A)** Isolated flies no longer significantly differ from group-housed flies in their walking distance or speed 10-15min after entry into the arena. Graphs mean ± SEM. **(B)** Change in walking distance of each fly between video 1 and video 2. Paired t-test (Males: grouped t=4.716, df=9, p=0.0011; isolated t=2.212, df=9, p=0.0543; Females: grouped t=2.836, df=9, p=0.0159). *p<0.05, **p<0.01. **(C)** Group-housed and isolated flies are not different in their spatial distributions within an arena in video 2. Graphs mean ± SEM. Please note that there was a technical error when obtaining video 1 from one of the isolated females (hence the discrepancy in sample numbers between video 1 and 2 for this group).

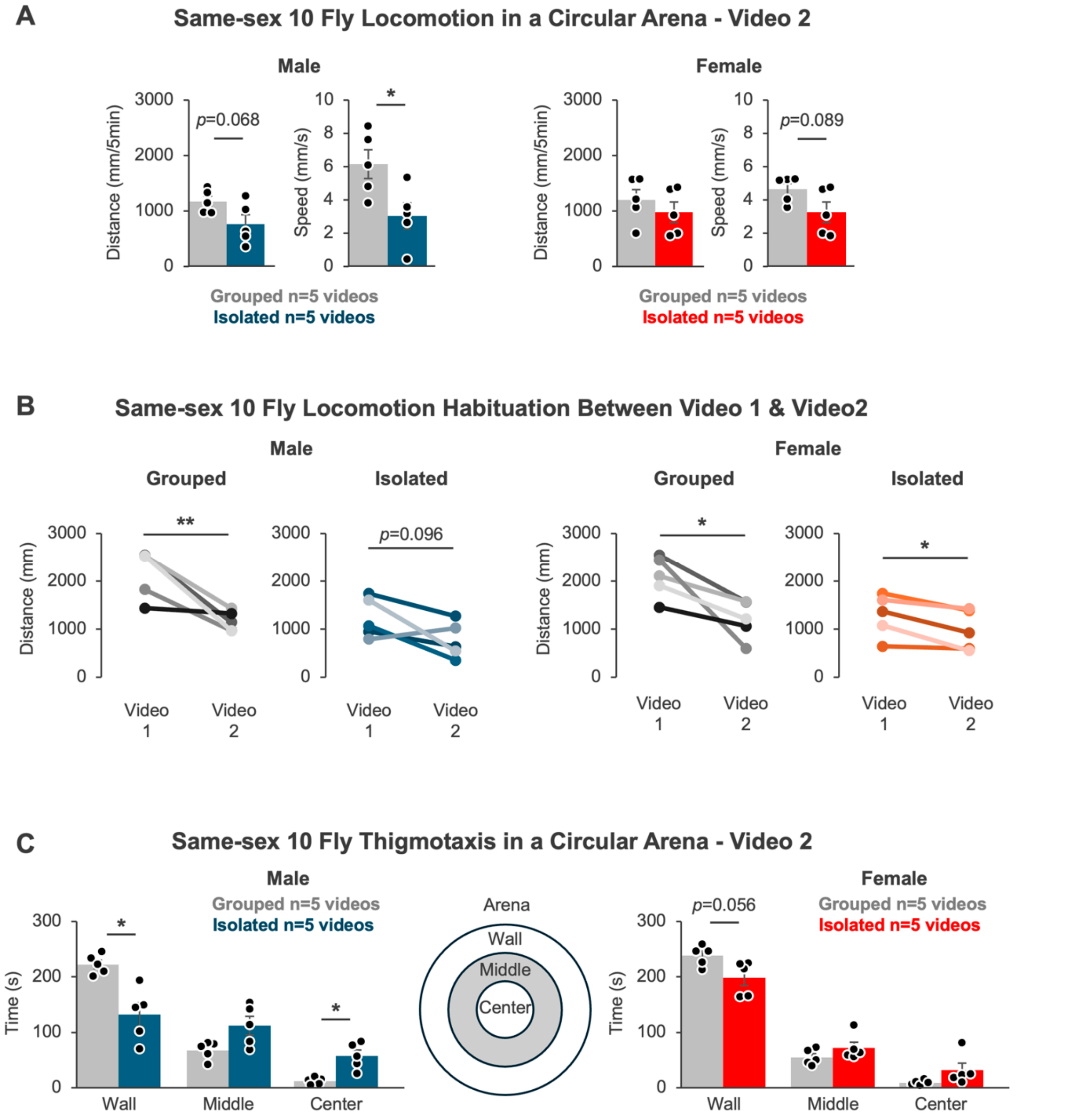

Figure S2. Locomotion of 10 same sex flies within an arena after 10min of habituation. **(A)** Isolated male flies display reduced walking speed compared to group-housed flies 10-15min after habituation to the arena. Unpaired t-test (t=2.670, df=8, p=0.0283). Graphs mean ± SEM, *p<0.05. **(B)** Change in walking distance in 10 fly videos between video 1 and video 2. Paired t-test (Males: grouped t=3.936, df=4, p=0.017; Females: grouped t=3.449, df=4, p=0.0261; isolated t=3.556, df=4, p=0.0237). *p<0.05, **p<0.01. **(C)** The spatial distribution of group-housed and isolated flies within the FlyBowl arena differs when in 10 same sex fly groups, with isolated flies preferring center parts of the arena compared to the wall in video 2. Unpaired t-test with Bonferroni correction (Males: wall t=4.003, df=8, adjusted p=0.0117; center t=4.086, df=8, adjusted p=0.0105). Graphs mean ± SEM, *p<0.05, **p<0.01.

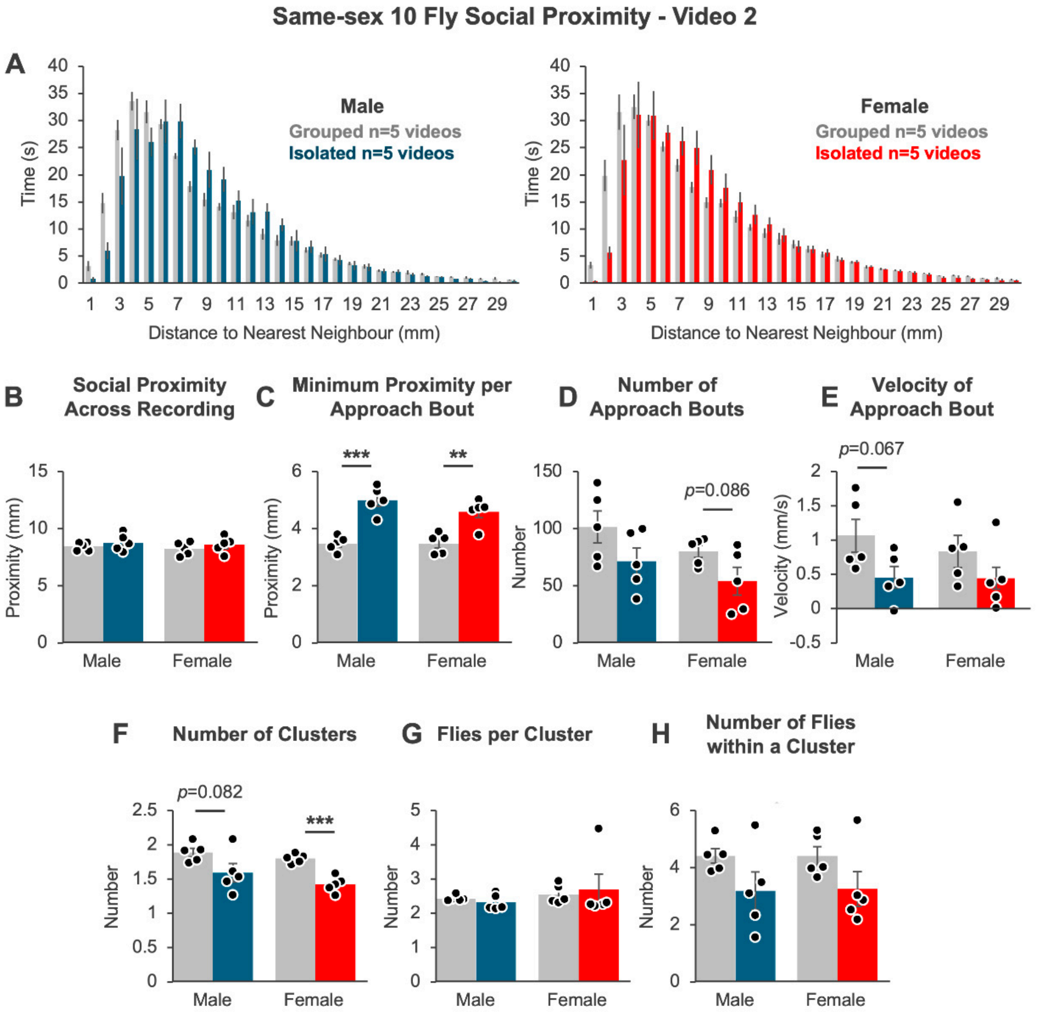

Figure S3. Measures of social proximity in group-housed and isolated flies after 10min of habituation. Measures associated with social proximity and social clustering during video 2. **(A)** Time spent in 1mm bins of proximity to a fly's nearest neighbour during video 2. **(B)** Average social proximity across video 2 recording is not different between grouop-housed and isolated flies. **(C)** Average approach bout minimum proximity is significantly higher in isolated flies compared to group-housed flies. Unpaired t-test (Males: t=6.294, df=8, p=0.0002; Females: t=4.145, df=8, p=0.0032). **(D)** Number of approach bouts performed by group-housed and isolated flies was not statistically significant in video 2. **(E)** There was no significant difference in average velocity of approach bouts between group-housed and isolated flies in video 2. **(F)** Female isolated flies continued to form significantly fewer social aggregation clusters than female group-housed flies in video 2. Unpaired t-test (Females: t=6.184, df=8, p=0.0003). **(G)** There were no differences in the numbers of flies per cluster between group-housed and isolated conditions in video 2. **(H)** There were no significant differences in the numbers of flies identified to be in a cluster in video 2. All graphs mean ± SEM, **p<0.01, ***p<0.001, ****p<0.0001.
